# The Diseases of the Heart and Aorta

**Published:** 1855-11

**Authors:** 


ART. IX.—*The Diseases of the Heart and Aorta.* By WILLIAM STOKES, Regius Professor of Physic in the University of Dublin; author of the "Treatment and Diagnosis of Diseases of the Chest," etc. Philadelphia: Lindsay & Blakiston. 1855.

We have not found time to examine critically, and do justice to what seems, from a casual glance at its contents, to be a very valuable work. The author (well known to our readers as the writer of a most useful work on Diseases of the Chest) has thrown out this book to the profession as embodying the clinical observations of more than a quarter of a century. It is not arranged on the numerical plan, neither does it embody all his cases, but it is to be taken as an effort on his part to express opinions resulting from observation and long experience, even though he is not in every instance able to refer that opinion to the facts on which it was originally founded.

To adopt his own language "The work is not intended as a full treatise on Cardiac Pathology, nor yet on Physical Diagnosis, but it aims at the rational application of these branches of knowledge to practical medicine."

While we are not authorized of our own knowledge to fully endorse this volume, we doubt not that it is really a valuable addition to practical knowledge of heart disease, and as such we feel safe in commending it to our readers.

